# Egocentric Dominance in Spatial Representations: The Role of Environmental Familiarity in Building Cognitive Maps

**DOI:** 10.1002/brb3.71393

**Published:** 2026-04-14

**Authors:** Shuting Lin, Senning Zheng, Yidan Qiu, Xiaoyu Zheng, Shuxin Jia, Taihan Chen, Ruiwang Huang

**Affiliations:** ^1^ School of Psychology, Center for Studies of Psychological Application, Guangdong Key Laboratory of Mental Health and Cognitive Science South China Normal University Guangzhou Guangdong China; ^2^ School of Education and Psychology, Institute of Applied Psychology, Fujian Province, Key Laboratory of Applied Cognition and Personality Minnan Normal University Zhangzhou Fujian China

**Keywords:** entorhinal cortex, parahippocampal place area, retrosplenial complex, spatial reference frame, spatial representation

## Abstract

**Background:**

Mental representations of spatial layouts and object locations are essential for building cognitive maps, which primarily rely on allocentric and egocentric reference frames. However, it remains unclear which reference frame individuals prefer for spatial processing, and how environmental familiarity may modulate these representations.

**Methods:**

Following Marchette et al. (2014), we conducted a longitudinal fMRI study in which subjects freely explored a virtual park and memorized the locations of 32 objects distributed across four museums under low and high familiarity conditions. During the fMRI scanning, the subjects performed a spatial judgment task to study how the brain constructs spatial reference frames.

**Results:**

Behavioral results revealed a consistent preference for an egocentric reference frame for spatial processing, regardless of environmental familiarity. Moreover, egocentric representations were more influenced by location than direction, especially in less familiar environments.

**Conclusion:**

The analysis of fMRI data showed that the retrosplenial complex (RSC) and parahippocampal place area (PPA) supported egocentric encoding. Although no allocentric encoding effect was observed, the entorhinal cortex (EC) exhibited significant sex differences in neural pattern similarity in the fully familiar condition, with males showing higher similarity than females. These findings suggest a robust egocentric cognitive map and underscore the need to consider individual and environmental factors in understanding how cognitive maps are constructed.

## Graphical abstract

Through virtual park exploration and fMRI, this study shows humans favor egocentric frames in spatial processing. RSC and PPA support egocentric encoding, while EC exhibited significant sex differences in neural pattern similarity in the fully familiar condition, highlighting individual and environmental influences on cognitive map construction.

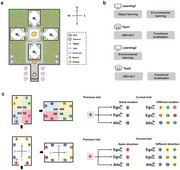

## 1 Introduction

Imagine arriving in Guangzhou city for the first time; you might easily remember specific landmarks such as the Canton Tower, the Guangdong Museum, or the tree‐lined Yuexiu Park. However, understanding how these landmarks connect within the larger spatial layout can be challenging. Effective spatial navigation requires individuals to form elaborate cognitive maps that represent object locations and spatial layouts. These maps are based on spatial reference frames (RFs), which consist of a reference point and a direction, allowing us to represent any location in a space. Accumulating evidence indicates that RF plays a key role in spatial cognition, especially in goal‐directed actions, spatial memory, and navigation (Bharmauria et al. 2025; Fiehler and Karimpur 2023; Jacob et al. 2017; Peer and Epstein 2021). Spatial RFs are commonly categorized as allocentric or egocentric (Colombo et al. 2017; Li et al. 2021; Ruggiero et al. 2021; Ruotolo et al. 2019). Allocentric RFs encode spatial relationships between objects based on a cardinal coordinate system (Clark et al. 2018; Julian et al. 2018), while egocentric RFs are based on the observer's perspective, adapting as they move through the environment (Filimon 2015; Lester and Dassonville 2014; Vukovic and Shtyrov 2017). In novel environments, egocentric cues often provide fast, practical reference points, enabling efficient navigation (Muffato and Meneghetti 2020; Peer and Epstein 2021; Weisberg et al. 2014). Over time, as individuals become familiar with their environments, they tend to shift toward allocentric strategies, which store spatial relationships more globally (Epstein et al. 2017; Schinazi et al. 2013). However, it remains unclear how environmental familiarity influences the preference for egocentric or allocentric RFs, particularly when processing direction and location.

Several studies showed that environmental familiarity plays a significant role in shaping spatial representations (Meneghetti et al. 2017; Merriman et al. 2016; Muffato et al. 2020) and can influence the corresponding neural activity patterns (Barry et al. 2012; Jafarpour and Spiers 2017; Shine et al. 2016). Woollett and Maguire (2011) suggested that environmental familiarity may influence allocentric spatial processing. In contrast, Merriman et al. (2016) suggested that environmental familiarity primarily influences egocentric spatial processing but not allocentric spatial processing. Additionally, individual differences, such as sex, may also impact spatial RF preferences. Previous studies showed that males tend to represent spatial information in a more allocentric fashion (De Goede and Postma 2015), whereas females tend to represent spatial information in a more egocentric fashion (Ycaza Herrera et al. 2019; Nazareth et al. 2019). Thus, we aimed to determine whether sex differences influence the way individuals use egocentric or allocentric RFs across different levels of environmental familiarity.

These two reference frames, egocentric and allocentric, are supported by different brain regions. Allocentric and egocentric neural representations of space include several brain areas, such as the retrosplenial cortex (RSC), parahippocampal place area (PPA), hippocampus (HPC), and entorhinal cortex (EC). The human HPC has long been implicated in allocentric spatial processing (Doeller et al. 2008; Iglói et al. 2010; Rodriguez 2010), encoding an object's position relative to other objects and environmental geometry (Guderian et al. 2015; Kumaran and Maguire 2005; Suthana et al. 2009). Grid cells in the EC encode global representations of the space, and their firing patterns become more refined with increasing experience, providing a neural basis for the allocentric framework (Carpenter et al. 2015; Killian et al. 2012; LaChance and Hasselmo 2024; Wernle et al. 2018). Human studies using fMRI and electrophysiological techniques have further confirmed the critical role of the EC in allocentric spatial representation (Chadwick et al. 2015; Jacobs et al. 2013; Julian et al. 2018; Shine et al. 2019). The RSC is involved in spatial memory and orientation (Alexander et al., 2023; Clark et al. 2018; Vukovic and Shtyrov 2017), particularly in relation to egocentric RFs, encoding a stable sense of orientation across environments with similar structures (Marchette et al. 2014). Additionally, the RSC contains head direction cells that respond to local features such as walls and corners (Alexander et al. 2020; Baumann and Mattingley 2010; LaChance and Hasselmo 2024; van Wijngaarden et al. 2020), reinforcing that the RSC processes spatial information relative to egocentric RFs. Moreover, the PPA is involved in processing egocentric representations, encoding spatial information based on the observer's perspective (Epstein et al. 2007; Henderson et al. 2008; Kunz et al. 2021; Weniger et al. 2010). However, despite extensive research on their functions, it remains unclear how environmental familiarity influences the preference for these RFs, especially when navigating novel and familiar environments.

In the current study, we conducted an experiment over four sessions spread across 2 weeks to investigate how the brain constructs RFs at different levels of environmental familiarity. By adapting the paradigm in Marchette et al. (2014), we recruited healthy subjects to explore a virtual environment freely and memorize the location of 32 objects distributed across four museums, using either allocentric or egocentric RFs. We acquired fMRI data from the subjects twice in 2 weeks: (1) when they were less familiar with the virtual park less than 2 days after the training, and (2) when they had become fully familiar with the park at 1 week after the training. During the fMRI scanning, the subjects performed a spatial judgment task in which they needed to judge whether a target object was located to the left or right of a to‐be‐remembered reference object. We hypothesized that the subjects would predominantly rely on egocentric RFs in less familiar environments and shift toward more allocentric RFs with an increase in environmental familiarity. Based on the behavioral data, we examined how environmental familiarity influences the reference system when processing spatial representations. We performed a regions of interest (ROI)‐based multivoxel pattern analysis (MVPA) to examine which brain areas contribute to the formation of these RFs and how they are modulated by environmental familiarity. Specifically, the MVPA was carried out on each of four ROIs, the parahippocampal place area (PPA), retrosplenial cortex (RSC), hippocampus (HPC), and entorhinal cortex (EC), which are known to be involved in allocentric or egocentric spatial processing.

## 2 Materials and Methods

### 2.1 Subjects

Twenty‐five healthy, right‐handed individuals from South China Normal University (SCNU) participated in this study. All had normal or corrected‐to‐normal vision and no history of neurological or psychiatric disorders. One subject quit the experiment, and another was excluded due to poor performance in the task, resulting in 23 subjects (11 F/12 M, mean age 21.6 ± 1.8 years old). This study was approved by the Institutional Review Board (IRB) of SCNU. All the subjects gave written informed consent in compliance with procedures approved by the IRB of the SCNU.

### 2.2 Virtual Environment

**FIGURE 1:**
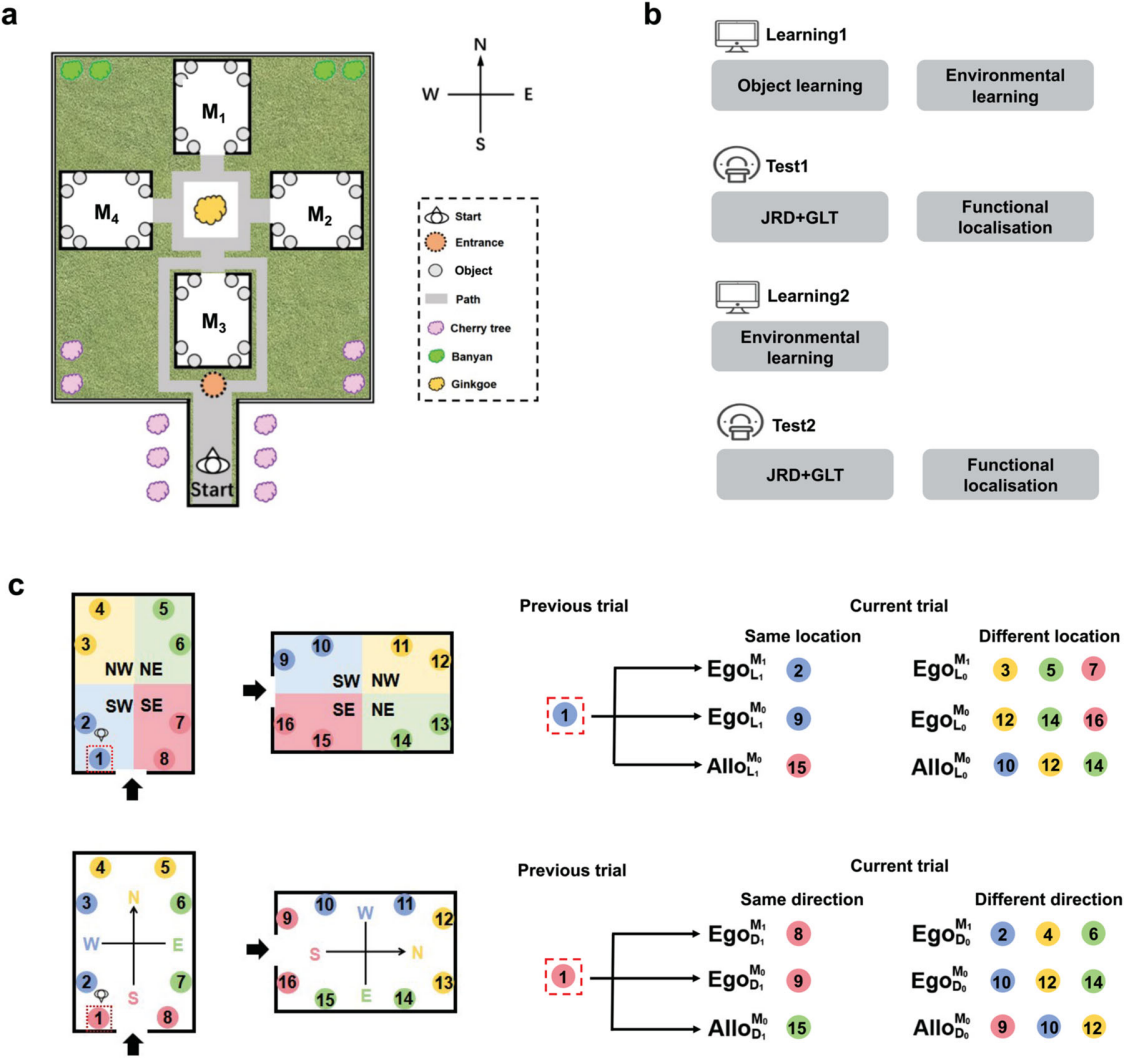
Experimental design and spatial attributes of priming effects based on the reference frames (RFs). (a) A bird's eye view of the virtual park, showing the positions of the four museums (M_1_–M_4_). Each museum was situated at different orthogonal directions with respect to the park. The letters N, E, S, and W indicate the allocentric directions of the park. Thirty‐two objects were displayed on pedestals throughout the park, represented by gray circles. The cherry, banyan, and ginkgo trees are landmarks in the park, serving as allocentric cues. (b) Experimental procedure. The experiment is a longitudinal design. The first learning session (Learning1, outside the scanner) comprised object learning and environmental learning. The first fMRI scan session (Test1) involved the judgment of relative direction (JRD) task, the global location task (GLT), and a 1‐back task for functional localization. The second learning session (Learning2) took place approximately 1 week after the first fMRI scan session, followed by the second fMRI scan session (Test2) less than 2 days. For full details on the procedure and tasks, see Methods. (c) Spatial attributes of priming effects. The bold arrow represents the entrance to the museum. Top row: Colored areas represent the four distinct egocentric locations: northeast (NE), northwest (NW), southeast (SE), and southwest (SW). The dashed box marks the subject's imagined viewpoint in the previous trial. The right side of the top row shows the priming effects of facing location, indicating whether the objects from the previous trial and the current trial had the same or different spatial locations. There are six possible combinations for location. For example, EgoM1 L1 represents a trial pair where the current object is in the same egocentric location and within the same museum as the previous trial. Bottom row: Colors indicate four egocentric directions. The right side of the bottom row displays the priming effects of facing direction, showing whether the object from the previous trial and the current trial have the same or different spatial directions in the same manner. There are six possible combinations for direction. For example, EgoM1 D1 represents a trial pair where the current object is in the same egocentric direction within the same museum as the previous trial. The numbers 1 and 0 indicate whether the attribute was the same or different. Allo, allocentric; D, direction; Ego, egocentric; L, location; M, museum.

The Source SDK Hammer Editor (http://www.valvesoftware.com, Valve Software, Bellevue, WA, USA) was used to create a virtual park, which was implemented in Counter‐Strike game software (http://www.valvesoftware.com). The virtual environment was displayed on a laptop screen. The subjects navigated the environment from a first‐person perspective using the mouse, while adjusting their direction with the arrow keys on the keyboard. Figure 1a shows the virtual park, which consisted of four identical large rectangular museums, various trees aligned along the north–south axis, and textured paths. Each museum had a unique appearance and was situated in one of the four orthogonal directions of the park, with an entrance connected to an internal carpeted path. Eight distinct, nameable objects were displayed in each museum. In total, thirty‐two different objects were located within the park. Specifically, the exhibited objects included animals, plants, tools, and household objects. To control for non‐spatial effects on spatial memory, we conducted a preliminary screening for the placement of the exhibited objects and developed two maps with identical spatial layouts. The only difference between the maps was the placement of the objects (Figure S1).

### 2.3 Experimental Procedure

Figure 1b illustrates the pipeline of the study, which was a longitudinal experimental design conducted over four sessions spread across 2 weeks: (1) the first learning session, which included object learning, environmental learning, and a judgment of relative direction (JRD) task, which was done outside the scanner; (2) the first fMRI scan session, which included six runs of JRD and global location tasks (GLT) as well as a run of a functional localization task; (3) the second learning session, which was identical to the first learning session; and (4) the second fMRI scan session, which was identical to the first test session. There was an interval of less than 2 days between each learning and test session. The second learning session took place approximately 1 week after the first test session. The first fMRI scan session was defined as the less familiar stage, and the second fMRI scan session was defined as the fully familiar stage.

#### 2.3.1 Object Learning: A Simple Matching Test

The test was presented on a laptop screen. The subjects were first asked to familiarize themselves with the names and appearance of objects sequentially located in the virtual park. The subjects were given enough time to remember the names and appearance of all the stimuli. After memorization, the subjects completed a simple matching test with 32 trials. In each trial, an object name was shown, and the subjects had 5 s to identify the correct corresponding object appearance from three options. The subjects’ learning performance was characterized by the number of errors. If the subjects made more than one error, the experimenter explained the task rules again and the subjects were asked to complete the same learning session again, until they made no more than one error.

#### 2.3.2 Environment Learning

The subjects explored the virtual environment freely from a first‐person perspective using the keyboard arrow keys. They were told to visit all four museums at least once. After 10 min of free exploration, the subjects were asked to perform a guided learning task for approximately 15 min. The task was carried out using two desktops. One screen displayed the target object, while the other showed the virtual environment for navigation. The subjects were presented with one target object at a time on the presentation screen and instructed to navigate to that object within the virtual environment. This setup allowed the subjects to view the target object on one screen while simultaneously exploring the environment on the other, ensuring that the indicator did not interfere with their movement or sense of spatial orientation. The objects were presented in a pseudo‐random order, with the constraint that two consecutive objects were always located within the same museum. The trials were organized into eight blocks, each containing eight trials. Within each block, two objects from the same museum were presented consecutively, and the order of the museums presented to the subjects was randomized. After locating the eight objects in a block, the subjects returned to the entrance of the park for a self‐paced break before starting the next block. Each object was located twice, resulting in a total of 64 trials. This design enabled the subjects to explore multiple museums within the virtual environment, while reinforcing their memory of the spatial relationships between objects within the same room, as well as the global layout of the environment.

#### 2.3.3 Judgment of Relative Direction Task

In each trial of the JRD task, the subjects imagined themselves standing in front of a reference object and responded via keyboard “press” buttons whether a target object was to the left or right of that imagined viewpoint. The objects used in the JRD task belonged to four categories: animals, plants, tools, and furniture, with eight objects in each category. During the JRD task, the reference object name was shown in yellow, and the target object name in white, centered on a black background. The target object was always located within the same museum as the reference object. The subjects had 4.8 s to respond, followed by a 1.2 s inter‐trial interval. All the objects from the four museums were used in the behavioral post‐tests. Each subject was required to achieve a threshold in the JRD response accuracy (RA) score: 60%–70% in the first learning session and over 90% in the second learning session. If they did not achieve the required RA, they were asked to complete another learning session with the same stimuli.

For the fMRI sessions, stimuli were presented using a projector–mirror–screen system. The subjects viewing the screen via a mirror mounted on the head coil were able to see stimuli displayed on a rear projection screen positioned at the head end of the scanner bore. Each subject attended the task‐fMRI experiment, which consisted of two scan sessions. Each fMRI scan session lasted about 40 min and included six runs. Each run included 64 JRD trials and 4 GLT trials. Similar to Marchette et al. (2014), we selected half of the objects as stimuli in the first fMRI scan session. The objects from the remaining two museums were presented in the second fMRI scan session, ensuring that all 32 objects were covered across the two fMRI scan sessions. The stimuli were chosen from orthogonally aligned museums, such as M_1_–M_2_, M_2_–M_3_, M_3_–M_4_, or M_4_–M_1_ (Figure 1a). Each fMRI run lasted about 5 min, and each trial was displayed for 4.8 s. If the subjects responded within the response window, the trial screen remained visible until the end of the response window, and the next trial began immediately. After the subjects completed each fMRI run, a self‐paced break was given before continuing. Responses were recorded using a 4‐button bimanual button box. Specifically, the subjects pressed the “1” key for the judgment of left and the “4” key for right. No feedback was provided during the JRD task. In the GLT task, only the name of the object was displayed on the screen, and the subjects were required to identify the museum (North, East, South, or West) where the object was located. They were instructed to press the corresponding key based on the object's location: “1” for the North museum, “2” for the East museum, “3” for the South museum, and “4” for the West museum. Each trial lasted for 4.8 s, followed by a 1.2 s inter‐trial interval before the next trial began. The accuracy rates for the GLT task (62.36% for less familiar vs. 90.22% for fully familiar environments) suggested that subjects were able to recall allocentric representations of the environment more accurately with an increase in familiarity.

#### 2.3.4 Functional Localization Task

In addition to the main experiment, the subjects completed a functional localizer fMRI scan to define functional areas, specifically the PPA and the RSC (Julian et al. 2012). During the task, the subjects viewed a picture of outdoor scenes, objects, or scrambled objects in a random order. All images were carefully selected from publicly available online resources. The localization task consisted of 5 blocks of outdoor scenes, 5 blocks of object images, and 5 blocks of scrambled object images, with each block containing 16 trials, totaling 240 trials. Each trial was presented for 600 ms, with a 400 ms inter‐trial interval. The subjects were asked to press a button to report the 1‐back repeated pictures. This task ensured that subjects maintained focus throughout the localization task. At the start and end of the task, the subjects viewed a fixation cross “+” for 12 s without responding. The entire localization task lasted about 5 min.

### 2.4 Linear Mixed‐Effects Modeling for Behavior Analysis

Linear mixed‐effects models (LMMs) were applied to analyze the subjects’ RA and behavioral priming effects within the allocentric and egocentric RFs for both less and fully familiar environments. For the egocentric RF, we defined direction by the subject's initial facing direction when they entered each museum. For example, “local northwest” referred to facing the left‐hand corner opposite the doorway, and “local north” referred to facing the wall directly opposite the doorway in each museum (Figure 1c). For the allocentric RF, we defined direction according to the park's cardinal coordinate system (i.e., North, East, South, and West) across all museums and determined the location according to the park's secondary coordinates (i.e., Northeast, Northwest, Southeast, and Southwest). Specifically, we set up three LMMs for analyzing the behavioral data. Since male subjects performed more accurately than females in the fully familiar stage (96.70% vs. 92.99%, *t* = 2.131, *p* = 0.045), we included sex as a fixed‐effect in the LMMs, along with subject and run intercepts as random effects in the LMMs. The LMMs were implemented in *R* using the lmerTest package (Kuznetsova et al. 2017).

LMM1 (Equation 1) was used to examine the subjects’ preferences for RFs across different levels of environmental familiarity. Fixed effects included environmental familiarity (less familiar or fully familiar) and position (egocentric or allocentric than the preceding trial). Following the Marchette et al. (2014), we adopted a priming logic to dissociate spatial representations between consecutive trials. The egocentric position condition referred to the paired objects that maintained the same location and direction based on the subjects’ perspective. For example, Objects 1 and 9 in Figure 1c were placed in different museums but maintained the same spatial framework from the subject's perspective. In the allocentric position condition, the paired objects had the same location and direction based on the park's cardinal coordinates, but were placed in different museums. For example, Objects 1 and 15 in Figure 1c.

The egocentric condition referred to trials where both local location and direction were the same as in the preceding trial, but with objects in different museums. The allocentric condition involved trials in which both the location and direction, based on the park's cardinal coordinates, were the same as in the preceding trial, but with the objects in different museums. Trials with identical location and direction across consecutive trials were excluded because they primarily involved working memory rather than RFs. Therefore, the LMM1 was specified as follows:

(1) $$\begin{array}{ccc} & & \left(\mathrm{LMM}1\right):\mathrm{Accuracy}∼\;\mathrm{environmental}\;\mathrm{familiarity}\times \mathrm{position}+\;\\ & & \left(1|\mathrm{Subject}\right)+\left(1|\mathrm{Run}\right) \end{array}$$

Based on the LMM1 results, we further examined whether the subjects’ egocentric or allocentric representations relied on location or direction information. To do this, we constructed two separate linear mixed‐effects models, LMM2 and LMM3. In LMM2 (Equation 2), three predictors were introduced as the fixed effects of LMM2, including environmental familiarity, museum (same or different from the preceding trial), and location (same or different from the preceding trial). To ensure that priming effects were due to the location repetition rather than the direction repetition, we excluded trials in which the facing object in the preceding trial came from the same direction in the same museum or from an equivalent direction in a different museum (Figure 1c). In the LMM3 (Equation 3), we replaced the fixed effect of location with direction (same or different from the preceding trial). To ensure that the priming effects were due to the direction repetition rather than the location repetition, we excluded the trials in which the facing object came from the same location in the same museum or from an equivalent location in a different museum. The LMM2 and LMM3 were:

(2) $$\begin{array}{ccc} & & \left(\mathrm{LMM}2\right):\mathrm{Accuracy}∼\mathrm{environmental}\mathrm{familiarity}\times \mathrm{museum}\times \;\\ & & \mathrm{location}+\mathrm{Sex}+\left(1|\mathrm{Subject}\right)+\left(1|\mathrm{Run}\right) \end{array}$$

(3) $$\begin{array}{ccc} & & \left(\mathrm{LMM}3\right):\mathrm{Accuracy}∼\mathrm{environmental}\mathrm{familiarity}\times \mathrm{museum}\;\\ & & \;\times \mathrm{direction}+\mathrm{Sex}+\left(1|\mathrm{Subject}\right)+\left(1|\mathrm{Run}\right) \end{array}$$

### 2.5 MRI Data Acquisition

The MRI data were acquired on a Siemens Trio 3T MRI scanner with a 32‐channel head coil at the Brain Imaging Center of SCNU. The fMRI data were obtained using a multi‐band gradient‐echo EPI sequence with the following parameters: TR = 1,200 ms, TE = 41.6 ms, flip angle = 52°, slice acceleration factor = 5, data matrix = 88 × 88, FOV = (211 mm)^2^, slice thickness = 2.4 mm without inter‐slice gap, voxel size = (2.4 mm)^3^, phase encoding direction A >> P, and 65 transversal slices covering the whole brain. To correct for susceptibility‐induced geometric distortions and BOLD signal loss in the functional images, we also acquired a field map of the whole brain using a double‐echo gradient‐echo sequence with the following parameters: TR = 735 ms, TE1/TE2 = 5.04 ms/7.5 ms, flip angle = 60°, FOV = (211 mm)^2^, voxel size = (2.4 mm)^3^. In addition, high‐resolution brain anatomical images were acquired using a T1‐weighted 3D MP‐RAGE sequence with the following parameters: TR = 1,600 ms, TE = 2.98 ms, flip angle = 9°, slice thickness = 1 mm, FOV = (256 mm)^2^, data matrix = 256^2^, voxel size = (1.0 mm)^3^, and 176 sagittal slices covering the whole brain.

### 2.6 Functional MRI Analysis

#### 2.6.1 Functional Data Preprocessing

The fMRI data were preprocessed using fMRIPrep version 20.2.7 (Esteban et al. 2019) based on Nipype 1.6.1 (Gorgolewski et al. 2011). The fMRI data preprocessing pipeline included: (i) estimation and correction of head movement in six parameters, (ii) slice‐timing correction, (iii) correction of BOLD signal and geometric distortion with the field map, (iv) spatial realignment, co‐registration of brain structural and functional images, and (v) segmentation of brain structural images into white matter (WM) and gray matter (GM) maps with normalization into voxel‐size (2 mm)^3^ in MNI standard space. The fMRI data for the localizers were spatially smoothed with a Gaussian kernel of 6 mm full width at half maximum (FWHM), whereas the experimental fMRI data were not spatially smoothed.

#### 2.6.2 Definition of Regions of Interest

The fMRI data from the functional localizer run were used to define the scene‐selective ROIs in each subject. Specifically, we conducted a general linear model (GLM1) analysis by using FSL/FEAT (Jenkinson et al. 2012) to identify the voxels that responded to the different scenes. Three regressors for scenes, objects, and scrambled objects were convolved with a double‐gamma HRF. The PPA and the RSC were defined within each subject's individual brain space as the regions responding more strongly to scenes than to objects (*p* < 0.0001, uncorrected; Epstein and Kanwisher 1998). To ensure that the ROIs in each hemisphere consisted of an equal number of voxels in all subjects, we chose the 100 voxels with the highest activity that fell within the scene‐selective parcels for the PPA and RSC (Julian et al. 2012). These hemisphere‐specific functional ROIs were then combined to create bilateral ROIs, resulting in 200 voxels per ROI. To further explore allocentric representations, we defined two additional ROIs, the hippocampus (HPC) and the entorhinal cortex (EC). The HPC was defined using the Harvard–Oxford subcortical structural atlas (Desikan et al. 2006) with a probability threshold > 90%. The EC was defined from the Jülich histological atlas (Jenkinson et al. 2012) with a probability threshold > 90%.

#### 2.6.3 ROI‐Based Multivoxel Pattern Analysis

We performed a GLM2 analysis for the subsequent multivoxel pattern analysis (MVPA). For each run of the fMRI data, we included 17 main regressors, 16 for the involved objects, and 1 for the GLT task. Six motion parameters were also included as nuisance regressors. Each regressor was convolved with a double‐gamma hemodynamic response function (HRF). The temporal derivative of each regressor was also included to correct for slice‐timing and timing delays in the HRF. The GLM2 was conducted for each of the 12 fMRI runs for each subject. In this way, we obtained 16 beta‐maps, each corresponding to the fMRI response to an object for each run and for each subject.

Following Marchette et al. (2014), we built four neural representational similarity matrices (RSMs) for location or direction based on allocentric and egocentric RFs. In this context, pattern similarity was defined as the Pearson correlation between multivoxel activation patterns extracted from independent data groups (i.e., odd vs. even runs). First, we extracted the 16 beta‐weights for each run of the fMRI data from subject‐specific ROIs. Second, the six runs for each fMRI session were divided into two groups, the odd and even runs. For each group, we created multivoxel activation patterns for each object by averaging the beta‐weights across runs and concatenating across all voxels within the ROI. Next, the cocktail mean pattern (mean activity in each voxel across all objects) was subtracted from the 16 objects. The resulting object‐specific patterns were then compared using Fisher *z*‐transformed Pearson correlations between even and odd runs to create a 16 × 16 matrix for each subject for the location or direction based on the different RFs. Following the logic of behavioral analysis, we examined the egocentric representations of location while controlling direction, and vice versa. To assess the neural effects of location, for example, we compared the pattern similarity for views facing the same egocentric location to the similarity for views facing different locations (Figure S2). For each ROI, the repeated‐measures ANOVAs were conducted to examine the effects of egocentric representation of location on neural pattern similarity using the pattern similarity correlation matrices as the dependent variable. The independent variables included museum (same vs. different), location (same vs. different), and environmental familiarity (less familiar vs. fully familiar). Similarly, for the egocentric representation of direction, we performed a repeated‐measures ANOVA in the same manner. For the allocentric representations, repeated‐measures ANOVAs were performed with direction (or location) and environmental familiarity as factors. Moreover, we conducted an additional post‐hoc exploratory analysis to examine factors that might influence allocentric representations. Specifically, we included sex as a between‐subjects factor in the analyses to explore possible sex differences in the use of spatial reference frames.

### 2.7 Visualization and Reconstruction of the Representational Spaces

To reconstruct the spatial layout of the museum within the ROIs, we first averaged the Pearson correlation matrices for the ROI‐specific neural similarity matrices across the subjects. These correlation matrices were averaged to produce a grand correlation matrix, which was submitted to multi‐dimensional scaling to produce a 2D map of the objects. The resulting map was subsequently aligned with the schematic of our museums using Procrustes analysis (Gower and Dijksterhuis 2004). To explore whether the RSC and PPA could reconstruct egocentric representations, we averaged the correlation matrices from those corresponding to the less and fully familiar stages separately. These matrices were converted into dissimilarity matrices by normalizing the values between 0 and 1, subtracting each value from 1. Next, we calculated the object layout matrix by computing the Euclidean distances between objects based on their actual physical locations within each museum. To assess the relationship between physical and neural spaces, we then correlated the off‐diagonal elements of these 8 × 8 distance matrices with the corresponding elements of the neural dissimilarity matrices from each ROI using Spearman's rank correlation. To assess the significance of these correlations, we performed a permutation test by randomly shuffling the values in each ROI's dissimilarity matrix and calculating the correlations between the shuffled matrix and the object layout matrix 10,000 times, to generate a null distribution of correlations. We then compared the actual correlation between the unshuffled dissimilarity matrix and the object layout matrix to this null distribution. Finally, we compared the dissimilarity matrices of the PPA and the RSC using a Wilcoxon rank‐sum test.

### 2.8 Statistical Analyses

All statistical analyses were performed on the whole subject group (*n* = 23). Behavioral data were analyzed using the LMMs implemented in *R* using the lmerTest package to assess the effects of reference frame (egocentric vs. allocentric), environmental familiarity (less vs. fully familiar), and sex on task accuracy. Subject and run were modeled as random intercepts. LMMs with Tukey‐adjusted post hoc pairwise comparisons were employed, with *p* < 0.05 considered significant. For the MVPA, RSC, and PPA were defined using the top 100 voxels per hemisphere based on localizer scans (scenes > objects), following previous studies, while the HPC and EC ROIs were anatomically defined using standardized structural atlases. To assess the statistical significance of the representational reconstructions, we conducted permutation tests (10,000 iterations), comparing actual correlations between ROI‐specific dissimilarity matrices and object layout matrices against null distributions obtained by random shuffling. Comparisons between ROIs (PPA vs. RSC) were performed using Wilcoxon rank‐sum tests (two‐tailed). To ensure reproducibility, we provide the analyzed, processed, and resulting source data in the repository. Data are available online upon reasonable request.

## 3 Results

### 3.1 Behavioral Results

**FIGURE 2:**
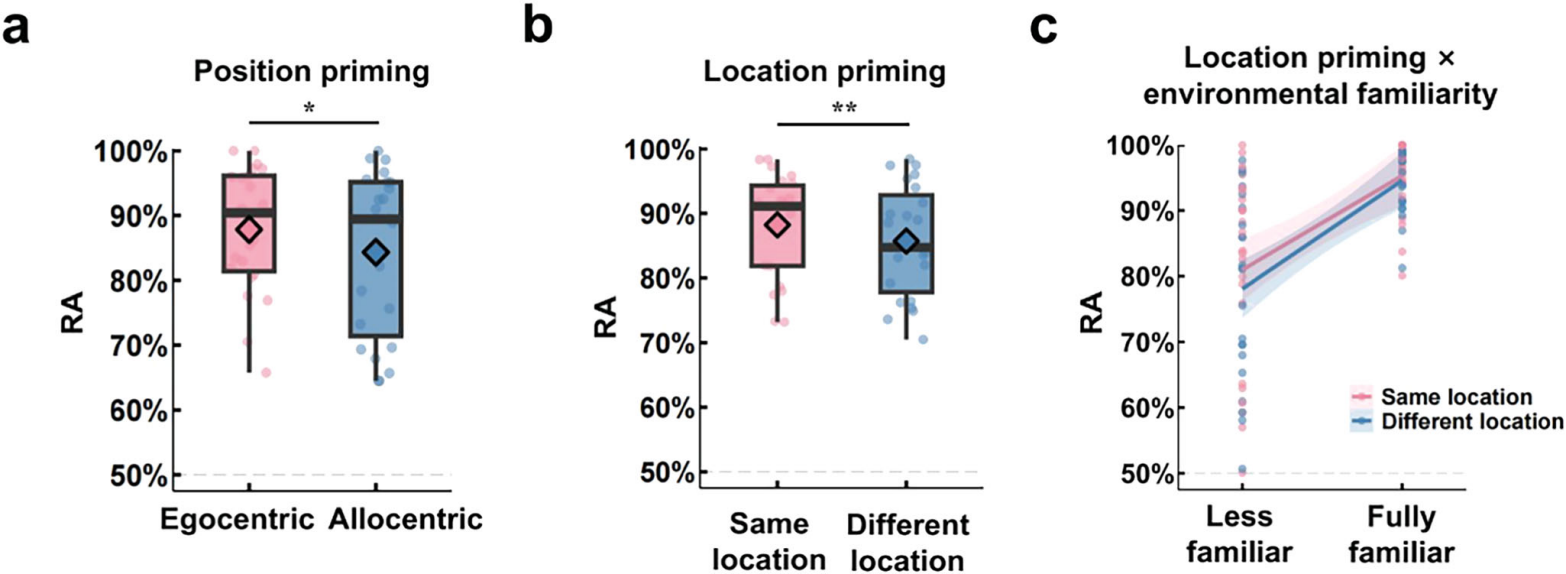
Priming effects of facing position and location on response accuracy (RA) in the judgment of relative direction (JRD) task. (a) Priming effect of facing position on RA. The subjects showed higher RA in the egocentric priming than in the allocentric priming, indicating a preference for using the egocentric RF in the spatial representation. Pink dots and boxplots represent the egocentric effects, while blue dots and boxplots represent the allocentric effects. Boxplot whiskers indicate the minimum to maximum values, horizontal lines inside the boxes indicate medians across the subjects, diamonds indicate the averages across all subjects, and boxes indicate values between the upper and lower data quartiles. Asterisks represent significant differences. (b) Priming effect of facing location on RA. The subjects responded more accurately when imaging objects consecutively within the same location than in different locations. Pink dots and boxplots represent the same location trials, and blue dots and boxplots represent the different location trials. *p < 0.05; **p < 0.01. (c) The interaction between location effects and environmental familiarity. The result showed that the RA gap between the same and different locations narrowed as environmental familiarity increased. The shaded areas indicate the 95% confidence intervals for RA across the subjects as a function of environmental familiarity. The dashed line at 50% represents the chance level performance, indicating the baseline RA expected if subjects were guessing randomly.

We first analyzed which type of RF is cognitively preferred in less familiar and fully familiar environments. The first linear mixed‐effects model (LMM1) revealed a significant main effect of position (*F* = 4.55, *p* = 0.033; Figure 2a), with a higher response accuracy (RA) for egocentric representation than for allocentric representation (egocentric vs. allocentric: 87.7% vs. 84.4%). The interaction between environmental familiarity and spatial reference representation did not reach significance (*F* = 1.06, *p* = 0.302). These results revealed that subjects consistently preferred using an egocentric RF, regardless of environmental familiarity.

Next, we explored whether the subjects’ egocentric representations relied on location or direction. The second linear mixed‐effects (LMM2) model revealed a significant main effect of location on RA (*F* = 6.97, *p* = 0.008; Figure 2b left), showing a higher RA when subjects faced the same location as in the preceding trial than when they faced a different location. The interaction between location and environmental familiarity reached marginal significance (*F* = 3.42, *p* = 0.064). Specifically, the RA was higher when the location was the same as in the preceding trial than when the location was different in the less familiar stage (same vs. different: 80.9% vs. 76.8%, *p* = 0.008). In contrast, in the fully familiar stage, no significant difference was found between the same and different locations (same vs. different: 95.3% vs. 94.5%, *p* = 0.943; Figure 4b). For the LMM3, neither the main effects of direction (*F* = 0.31, *p* = 0.575) nor museum (*F* = 0.45, *p* = 0.503) nor their interaction (*F* = 1.29, *p* = 0.257) on RA were significant. These results suggested that egocentric representations were more influenced by location than by direction, particularly in less familiar environments.

### 3.2 Multivoxel Pattern Analysis for Egocentric Reference Frame

**FIGURE 3:**
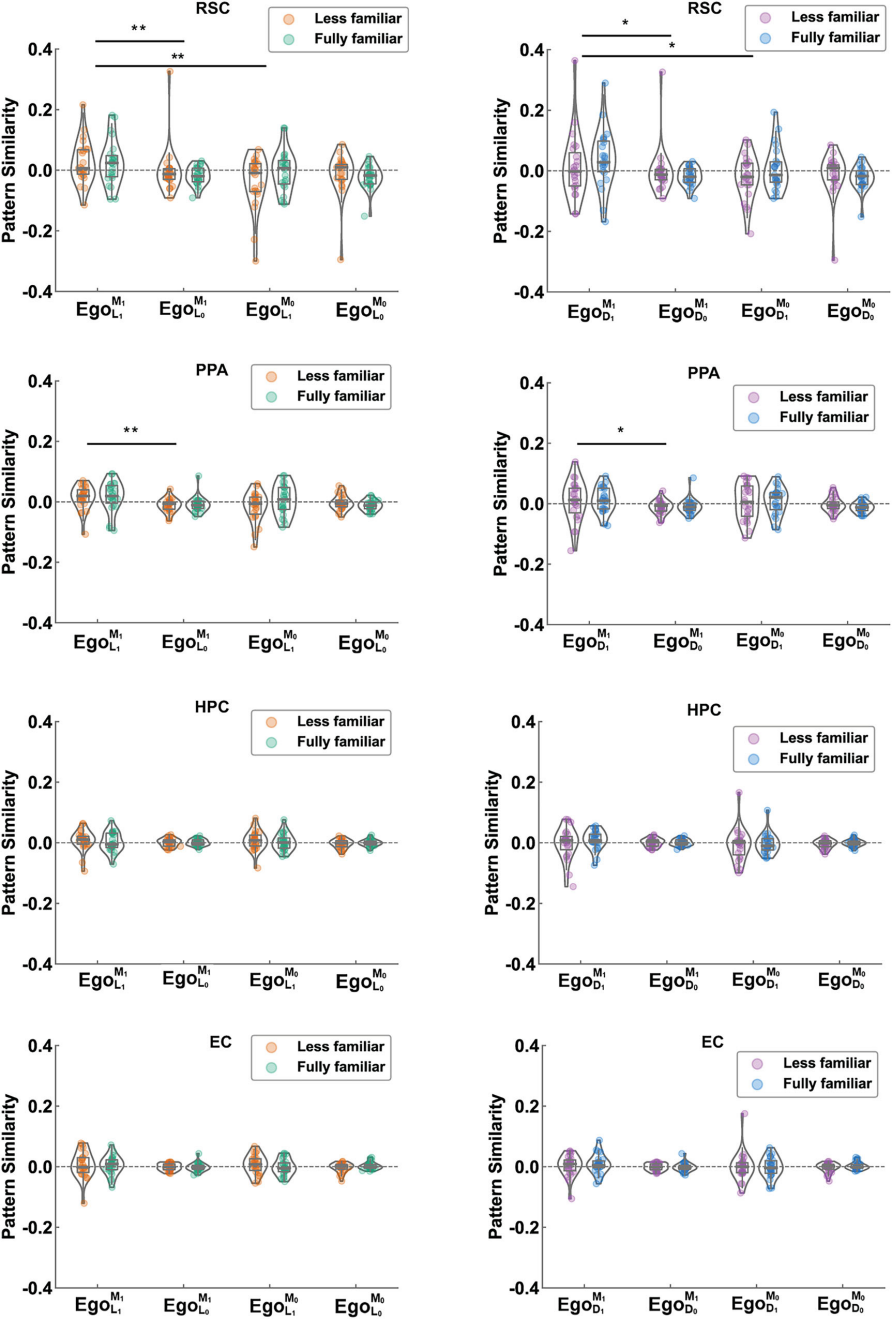
Neural pattern similarity for egocentric location and direction in four brain regions, the retrosplenial complex (RSC), parahippocampal place area (PPA), hippocampus (HPC), and entorhinal cortex (EC). Left column: Pattern similarity for the four egocentric location conditions. Right column: Pattern similarity for the four egocentric direction conditions. Each point represents one subject. *p < 0.05; **p < 0.01. The numbers 1 and 0 indicate whether the attribute was the same or different. Allo, allocentric; D, direction; Ego, egocentric; L, location; M, museum.

Figure 3 shows the results of the MVPA for encoding egocentric RFs. For each ROI, a 3‐level repeated measures ANOVA was performed to explore egocentric representations of location. For the HPC and EC, neither the main effects nor their interactions were significant. For the RSC, we found a significant main effect of museum, as well as a significant interaction between location and museum. Specifically, the main effect of the museum was significant in both the left RSC (*F* = 9.50, *p* = 0.005) and right RSC (*F* = 5.06, *p* = 0.035), with higher pattern similarity for objects in the same museum than different museums. The main effect of location encoding was significant in the left RSC (*F* = 4.70, *p* = 0.041) but not in the right RSC (*F* = 0.58, *p* = 0.456). The interaction between location and museum was significant in the left RSC (*F* = 8.67, *p* = 0.007) but not in the right RSC (*F* = 3.52, *p* = 0.074). A simple effect analysis showed that the interaction effect was driven by a significantly higher pattern similarity for the same location in the same museum than for different locations in the same museum (*p* = 0.002) or for the same location in different museums (*p* = 0.002). No significant difference was found between different locations in the same museum and different locations from different museums (*p* = 0.791). For the PPA, we found a significant main effect of location and a marginal interaction between location and museum (Table 1).

**TABLE 1:** Results of the MVPA for egocentric reference frame encoding across four regions, the retrosplenial complex (RSC), parahippocampal place area (PPA), hippocampus (HPC), and entorhinal cortex (EC).

	RSC	PPA	HPC	EC
**Egocentric representations of location**				
Location	*F* = 2.10, p = 0.161	F = 4.88, p = 0.038	F = 3.45, p = 0.077	F = 1.69, p = 0.207
Museum	F = 6.55, p = 0.018	*F* = 4.04, p = 0.057	F = 0.45, p = 0.511	F = 0.05, p = 0.820
Environmental familiarity	*F* = 0.07, p = 0.787	*F* = 1.23, p = 0.280	F = 0.35, p = 0.561	F = 0.05, p = 0.820
Location × museum	*F* = 6.19, p = 0.021	F = 3.96, p = 0.059	F = 0.08, p = 0.783	F = 0.13, p = 0.720
Location × environmental familiarity	*F* = 1.64, p = 0.214	*F* = 1.41, p = 0.248	F = 1.01, p = 0.326	F = 1.02, p = 0.325
Museum × environmental familiarity	*F* = 0.54, p = 0.468	*F* = 0.09, p = 0.768	F = 0.03, p = 0.854	F < 0.01, p = 0.956
**Egocentric representations of direction**				
Direction	*F* = 4.15, p = 0.054	*F* = 5.02, p = 0.035	F = 0.01, p = 0.914	*F* = 0.57, p = 0.457
Museum	*F* = 2.78, p = 0.109	*F* < 0.01, p = 0.957	F = 1.72, p = 0.203	*F* = 0.57, p = 0.459
Environmental familiarity	*F* = 1.10, p = 0.305	*F* < 0.01, p = 0.981	F = 1.57, p = 0.223	*F* = 0.34, p = 0.567
Direction × museum	*F* = 3.79, p = 0.064	*F* = 0.24, p = 0.630	F = 0.40, p = 0.533	*F* = 0.69, p = 0.415
Direction × environmental familiarity	*F* = 3.52, p = 0.074	*F* = 0.46, p = 0.506	F = 0.38, p = 0.540	*F* = 0.01, p = 0.911
Museum × environmental familiarity	*F* < 0.01, p = 0.933	*F* = 0.21, p = 0.652	F = 0.05, p = 0.816	*F* = 0.01, p = 0.903

Similarly, a 3‐level repeated measures ANOVAs was performed to explore egocentric representations of direction for each ROI. For the HPC and EC, neither main effects of direction nor their interactions were significant for egocentric representations of direction. For the RSC, we found a marginally significant effect of direction. The left RSC encoded egocentric information of direction (*F* = 7.07, *p* = 0.014, Figure S3) with an interaction between direction and museum (*F* = 4.67, *p* = 0.042). Another simple effect analysis indicated that the effect was driven by higher pattern similarity for the same direction in the same museum, more than for different directions in the same museum (*p* = 0.001) or for the same direction in a different museum (*p* = 0.001). No significant difference was found between the same and different directions in different museums (*p* = 0.654). No main effect or interaction was observed in the right RSC (all *ps* > 0.073). In the PPA, a significant main effect of direction encoding was found (Figure 3). Specifically, the left PPA revealed a significant main effect of direction encoding (*F* = 4.78, *p* = 0.040), but no main effect of direction (*F* = 3.42, *p* = 0.078), museum (*F* = 0.05, *p* = 0.829), environmental familiarity (*F* = 1.41, *p* = 0.248), or direction × museum (*F* = 0.20, *p* = 0.656), direction × environmental familiarity (*F* = 0.31, *p* = 0.580), or museum × environmental familiarity (*F* = 0.01, *p* = 0.907) were found in the right PPA (Table 1).

### 3.3 MVPA for Allocentric Reference Frame Encoding

We examined whether these brain regions are involved in encoding allocentric information related to location. There were no significant main effects of location or environmental familiarity, nor were their interactions significant for any ROI (all *ps* > 0.301). When analyzing allocentric direction information, neither the main effects of direction nor environmental familiarity were significant for the HPC (all *ps* > 0.744), nor were their interactions (*F* = 0.06, *p* = 0.803). Similarly, for the EC, neither the main effects of direction or environmental familiarity (all *ps* > 0.331) nor their interactions (*F* = 0.69, *p* = 0.415) were significant. Moreover, an independent sample *t*‐test in the less familiar stage revealed no sex differences in the allocentric processing of location and direction in any of the ROIs (all *ps* > 0.122). However, in the fully familiar stage, we observed that males had stronger allocentric direction coding in the EC than females (*t* = 2.78, *p* = 0.011). Further analysis revealed that this effect was significant in the right EC (*t* = 3.23, *p* = 0.004) but not in the left EC (*t* = 1.76, *p* = 0.093). Follow‐up analyses within the male group alone revealed no significant main effect of direction, suggesting the EC results represent a sex difference in neural patterns during the fully familiar stage, rather than functional involvement in encoding allocentric information.

### 3.4 Visualization of the Spatial Layout in the RSC and PPA

**FIGURE 4:**
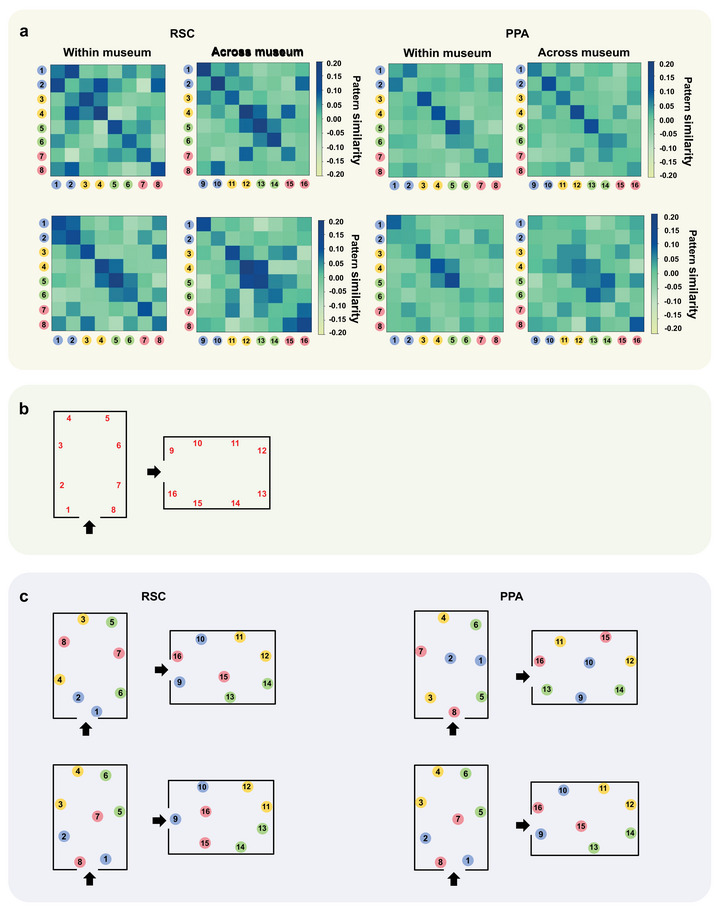
Reconstructing the spatial organization of the environment using multidimensional scaling (MDS) of fMRI pattern similarities. (a) Average the Pearson correlation matrices between objects within the same museum and across different museums in both the retrosplenial cortex (RSC) and parahippocampal place area (PPA). (Top row) Average matrix for the less familiar stage. (Bottom row) Average matrix for the fully familiar stage. (b) The red numbers indicate the real locations of the objects. (c) Reconstruction of museums from MDS and Procrustes alignment. The colored circles denote the four distinct egocentric locations, which are the result obtained from the MDS analysis. (Top row) for the less familiar stage. (Bottom row) for the fully familiar stage. Correlation matrices and the reconstructed spatial layouts for the entorhinal cortex (EC) and hippocampus (HPC) are provided in Figure S4.

Figure 4 shows the average correlation matrix and the reconstructed spatial organization of the environment in the RSC and PPA using multidimensional scaling (MDS). The coefficient of determination (*R* ^2^) was used to quantify the goodness of fit, representing the proportion of variance in the physical layout explained by the MDS‐reconstructed coordinates. We found that the spatial layouts derived from the RSC (*R* ^2^ = 0.63; *p* < 0.001) and from the PPA (*R* ^2^ = 0.61; *p* < 0.001) were similar to the actual layout of the museum. However, the spatial layout derived from the EC (*R* ^2^ = 0.20; *p* = 0.168) and HPC (*R* ^2^ = 0.30; *p* = 0.059) showed no significant correspondence with the museum's actual layout, suggesting that these regions may not contribute directly to encoding the egocentric spatial organization of the museum. Finally, no significant difference was found between dissimilarity matrices from the RSC and PPA (*z* = 0.35; *p* = 0.730). The results from these analyses showed that the information represented in the RSC and PPA was adequate for reconstructing a mental map of the local environment.

## 4 Discussion

In the current study, following the approach in Marchette et al. (2014), we employed a longitudinal design to study which spatial RFs individuals prefer for processing direction and location across different levels of environmental familiarity, as well as the neural mechanisms underlying these RFs. We found that subjects consistently preferred using egocentric RF regardless of environmental familiarity (Figure 2). Egocentric representations were more influenced by location than direction, particularly in the less familiar stage (Figure 2c). MVPA results indicated that both the RSC and PPA represented egocentric representations of location and direction (Figure 3). We also found that the EC may support the processing of allocentric information about direction in males during the fully familiar stage. Importantly, the HPC likely plays a key role in integrating and consolidating spatial information for building cognitive maps. Together, the RSC, PPA, and EC are essential for the flexible use of spatial representations in constructing and updating cognitive maps. Our study provides new insights into the neural mechanisms that underlie cognitive maps and guide the use of RFs, advancing our understanding of spatial navigation.

### 4.1 Dominance of the Egocentric RF in Spatial Representations

As suggested by Peer et al. (2021) and Bottini and Doeller (2020), learning an unfamiliar environment implies interpreting its spatial structure using RFs. These RFs are often selected based on cues such as an egocentric experience (Yamamoto and Philbeck 2013) and environmental properties (Marchette et al. 2017). By analyzing behavioral data, we found that the subjects’ RA improved when they consecutively judged objects sharing the same local spatial features, regardless of whether the objects were located in the same or different museums (Figure 2a). This result suggested that subjects relied on the egocentric RF, which aligns with prior research suggesting that egocentric representations are crucial for learning environments with similar spatial structures (Marchette et al. 2014). While subjects became fully familiar with the environment after additional learning, their reliance on egocentric RFs for spatial representation remained unchanged (Figure 2a). This suggests that familiarity did not significantly influence the selection of RFs in spatial representations. A possible explanation is that even with an increase in familiarity, the subjects continued to rely on egocentric RF because the judgment of relative direction (JRD) task required that they judge the relative directional relationship between objects within the same museum. In this situation, the subjects may not have needed to integrate allocentric strategies to form a comprehensive representation of the spatial environment because relying on local spatial cues alone provided enough information to perform the task. From the LMM analysis, we found that egocentric representations were more influenced by location than by direction, particularly in the less familiar stage (Figure 2c). In this task, the subjects were asked to determine whether one object was to the left or right of another, a judgment that is more directly tied to local location cues. In contrast, the direction‐based judgments often require integrating a broader spatial context, which might be more challenging in an unfamiliar environment where the local layout has not been fully internalized.

### 4.2 Spatial Representations of Egocentric RF in the PPA and RSC

We found that both the PPA and RSC played crucial roles in spatial representations involving egocentric RFs (Figure 3). This result was consistent with previous studies (Dilks et al. 2011; Jacob et al. 2017; Kim and Maguire 2018; Persichetti and Dilks 2019; Robertson et al. 2016). Berens et al. (2021) showed that after learning, different views of the same scene became more similar in the PPA and RSC. The MVPA showed that the PPA encodes egocentric information about location, with the activation patterns being more prominent in the left PPA (Figure S3). These findings align with the established role of the PPA in local scene perception (Epstein et al. 2007), particularly in its ability to encode different perspectives from the same location. Our results also showed that the left and right RSC appear to process different spatial information. Specifically, the left RSC was more involved in encoding the egocentric spatial information (Chadwick et al. 2015), such as the specific location within the environment, while the right RSC was more involved in encoding the spatial layout of the environment, such as the overall spatial context that distinguished one museum environment from another.

### 4.3 Spatial Representations of Allocentric RF in the HPC and EC

The current study found no significant allocentric encoding in any of the ROIs before accounting for sex differences. This raises two important questions: How do these ROIs contribute to allocentric spatial representations in different environments? And how do such representations evolve with an increase in familiarity? Unlike prior studies (Byrne et al. 2007; Huffman and Ekstrom 2019; Schinazi et al. 2013), our results showed no significant involvement of the HPC in allocentric processing. The HPC has long been implicated in the formation of allocentric spatial representations, particularly in tasks requiring navigation through novel environments. These tasks often rely on global spatial representations that integrate multiple spatial relationships, which likely engage the HPC. By contrast, we inferred that our task design emphasized the processing of spatial relationships on a local scale, without requiring the allocentric processing typically associated with the HPC. The subtlety of these neural effects is consistent with previous findings by Marchette et al. (2014), where robust behavioral priming observed in experiments did not consistently translate to significant neural effects during fMRI scanning. This suggests that priming‐based measures of allocentric representations might be less stable than those elicited by explicit task demands.

Regarding the EC, although no significant allocentric effects were observed, we found that neural pattern similarity was significantly modulated by sex in the fully familiar stage. The observed sex‐specific differences in the EC (specifically in the right EC) suggest that individuals might differ in their implicit processing of allocentric information. This indicates that such processing might be relatively highly dependent on individual factors, such as sex, even after an environment has become familiar. The emergence of these sex‐specific differences in the EC under the fully familiar stage may reflect the anchoring of specialized neurons, such as grid cells or boundary cells, to global environmental cues. Our findings highlight the EC as a dynamic and flexible system whose contribution to allocentric spatial representation is modulated by both internal (e.g., individual differences) and external (e.g., familiarity) factors. This underscores the importance of considering the role of the EC in mediating the integration of spatial information into coherent cognitive maps, particularly as environments become more familiar and structurally consolidated.

### 4.4 Sex‐Specific Neural Patterns in Fully Familiar Environments

In fully familiar environments, males showed a clear advantage in spatial knowledge retrieval. This result was consistent with previous findings that males tend to perform better in mental rotation tasks, which are often linked to allocentric spatial processing (Debarnot et al. 2013; Mochizuki et al. 2019). A meta‐analysis also found that males typically outperform females in spatial tasks involving mental rotation (Iachini et al. 2009a). These results indicate that environmental familiarity plays a role in modulating sex differences in spatial representations. No sex differences were observed in the EC during the less familiar environment. However, in the fully familiar stage, the males and females exhibited different neural activation patterns. Although no robust allocentric neural patterns was activated at the group level, males showed significantly higher pattern similarity for allocentric direction in the EC than females. This effect only emerged in the fully familiar environment, suggesting that environmental familiarity may modulate the sex‐related differences in spatial processing. The higher pattern similarity in the males may reflect a greater tendency or attempt to organize spatial information using an allocentric framework (de Castell et al. 2019; Harris et al. 2019; Iachini et al. 2009b). Conversely, the lower pattern similarity in the females might indicate a more persistent reliance on egocentric strategies. These findings indicate that in the absence of explicit task instructions, the brain's primary spatial processing mode appears to remain egocentric representations.

### 4.5 Limitations

The current study has several limitations. First, although we found that environmental familiarity had no significant effect on the selection of RFs in spatial representations, this result should be interpreted with caution. The training of our subjects only lasted for 2 weeks. This 2‐week training period was still much shorter than that of the familiarity that may typically occur over months or years in real‐world contexts. In light of this, we hypothesize that with long‐term exposure, individuals may gradually shift from egocentric representations toward allocentric ones, supporting the formation of more integrated and coherent cognitive maps. Second, we used different brain structural templates to define the ROIs which may introduce selection bias due to inter‐atlas variability in anatomical boundaries. In the current study, we defined the HPC according to the Harvard‐Oxford subcortical structural atlas (Desikan et al. 2006) and the EC according to the Jüelich histological atlas (Jenkinson et al. 2012). Such inter‐atlas variability in anatomical boundaries can affect both the size and spatial distribution of each ROI, potentially influencing the outcomes of the MVPA. Third, although the current study examined the neural mechanisms underlying allocentric and egocentric RFs separately, we did not analyze the transformation between the two RFs. Studies showed that transformations between allocentric and egocentric RFs involve the RSC, EC, and HPC (Alexander et al. 2023; Bicanski and Burgess 2018; Carpenter et al. 2015; Clark et al. 2018; Hartley et al. 2014). Future studies should explore how these regions contribute to the transformation between allocentric and egocentric representations, particularly with an increase in environmental familiarity. Fourth, it is important to acknowledge that the current study utilized an indirect, priming‐based measure of reference‐frame processing. Unlike traditional paradigms that require direct spatial identification, our task relies on the implicit facilitation of neural patterns across trials. While this design minimizes task‐driven biases, such indirect measures might exhibit lower statistical stability in capturing allocentric representations compared to explicit tasks. Consequently, the lack of significant allocentric processing in our scan should be interpreted with caution, as it may reflect the subtlety of implicit priming effects. Finally, the observed sex difference in allocentric representation within the EC was derived from a limited sample size. Therefore, this result should be interpreted cautiously due to potential statistical power constraints. The robustness of this result should be tested in future studies with a larger sample.

## 5 Conclusion

In conclusion, this study provides key insights into how spatial RFs are flexibly employed in human cognition. We found that individuals consistently preferred egocentric RFs for spatial processing, regardless of environmental familiarity. This result suggests that familiarity alone may not be sufficient to shift spatial encoding strategies, highlighting the need to explore other key factors beyond familiarity, such as individual differences or cognitive abilities. Additionally, we found that both the RSC and PPA supported the encoding of egocentric spatial information and that the EC was selectively involved in allocentric encoding. Moreover, we observed sex‐related differences in allocentric encoding under the condition of high familiarity, indicating a potential role of individual differences in shaping spatial representations. Collectively, these findings deepen our understanding of how the brain organizes spatial information into cognitive maps and underscore the practical importance of supporting spatial learning and orientation through effective navigation strategies in real‐world contexts.

## Author Contributions

**Shuting Lin**: conceptualization, investigation, methodology, formal analysis, writing – original draft, writing – review and editing. **Senning Zheng**: project administration, investigation, methodology, writing – review and editing. **Yidan Qiu**: validation, writing – review and editing. **Xiaoyu Zheng**: investigation, writing – review and editing. **Shuxin Jia**: writing – review and editing. **Taihan Chen**: writing – review and editing. **Ruiwang Huang**: conceptualization, resources, supervision, project administration, funding acquisition.

## Funding

This work was supported by funding from the Key Technologies R&D Program of Guangdong Province (Grant number: 2023B0303020002), National Natural Science Foundation of China (Grant numbers: 32371101 and 82171914), Guangdong Natural Science Foundation (Grant number: 2022A1515011022), striving for the first‐class, improving weak links and highlighting features (SIH) key discipline for psychology in South China Normal University, and National Key Research and Development Program of China (Grant number: 2018YFC1705006).

## Supporting information




**Supplementary figures**: brb371393‐sup‐0001‐figuresS1‐S4.docx

## Data Availability

The data that support the findings of this study are available from the corresponding author upon reasonable request.
