# Supplementary material for: Egocentric Dominance in Spatial Representations: The Role of Environmental Familiarity in Building Cognitive Maps
Source: Brain Behav. 2026 Apr 14;16(4):e71393. doi: 10.1002/brb3.71393 (PMC13080106; doi:10.1002/brb3.71393)
Supplement: Supplementary file 1 — Supplementary figures: brb371393‐sup‐0001‐figuresS1‐S4.docx [file BRB3-16-e71393-s001.docx]

**Egocentric dominance in spatial representations: The role of environmental familiarity in building cognitive maps**

Shuting Lin ^1 ¶^, Senning Zheng ^2 ¶^, Yidan Qiu^1^, Xiaoyu Zheng^1^, Shuxin Jia^1^, Taihan Chen^1^, Ruiwang Huang^1*^

^1^ School of Psychology, Center for Studies of Psychological Application, Guangdong Key Laboratory of Mental Health and Cognitive Science, South China Normal University, Guangzhou 510631, Guangdong, China.

^2^ School of Education and Psychology, Institute of Applied Psychology, Fujian Province, Key Laboratory of Applied Cognition and Personality, Minnan Normal University, Zhangzhou 363000, Fujian, China.

^¶^ equal contribution

^*^ Correspondence to

Professor Ruiwang Huang

School of Psychology

South China Normal University

Guangzhou, 510631, China.

Tel/Fax: +86 (0)20-8521 6499

Email: [ruiwang.huang@gmail.com](mailto:ruiwang.huang@gmail.com)

**Supplementary Materials**


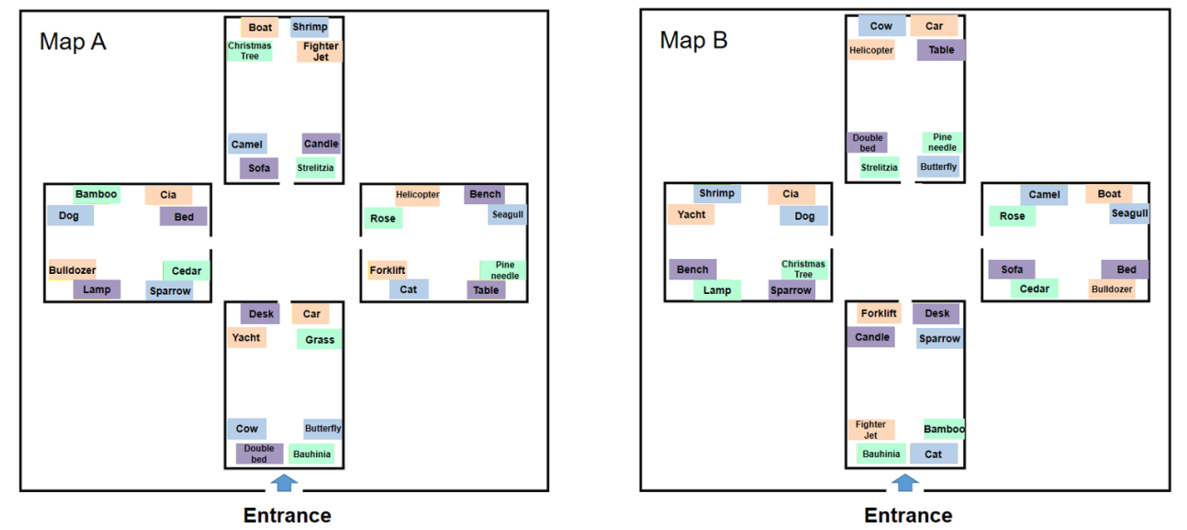
**Fig. S1** **Bird’s eye view of two maps (Map A and Map B) used in virtual environment.**

The two maps illustrate the layout of objects within the virtual park. Blue, green, orange, and purple correspond to the four categories, animals, plants, tools, and furniture. Except for the animal models, which had corresponding animation effects, all the other objects were static. To disassociate the spatial attributes of objects (such as direction and location) from the non-spatial attributes (such as category or similarity), we arranged the objects by following the principles: (1) Each museum contained objects from all of the four categories. (2) Objects from the same category were not placed in either the same direction or location within the museum. (3) Objects that were similar in appearance were not placed in the same museum (e.g., if a cat was placed in one museum, a dog was placed elsewhere). (4) There was not any local spatial overlap between the same object category (e.g., if a desk wais positioned on the left side of the entrance in one museum, no other museum will have a table in the same location). (5) No more than two objects from the same category shared the same local information in the four museums. To avoid non-spatial effects on spatial memory, we created two maps (Map A and Map B) with different object arrangements and randomly assigned the subjects to one of the maps.


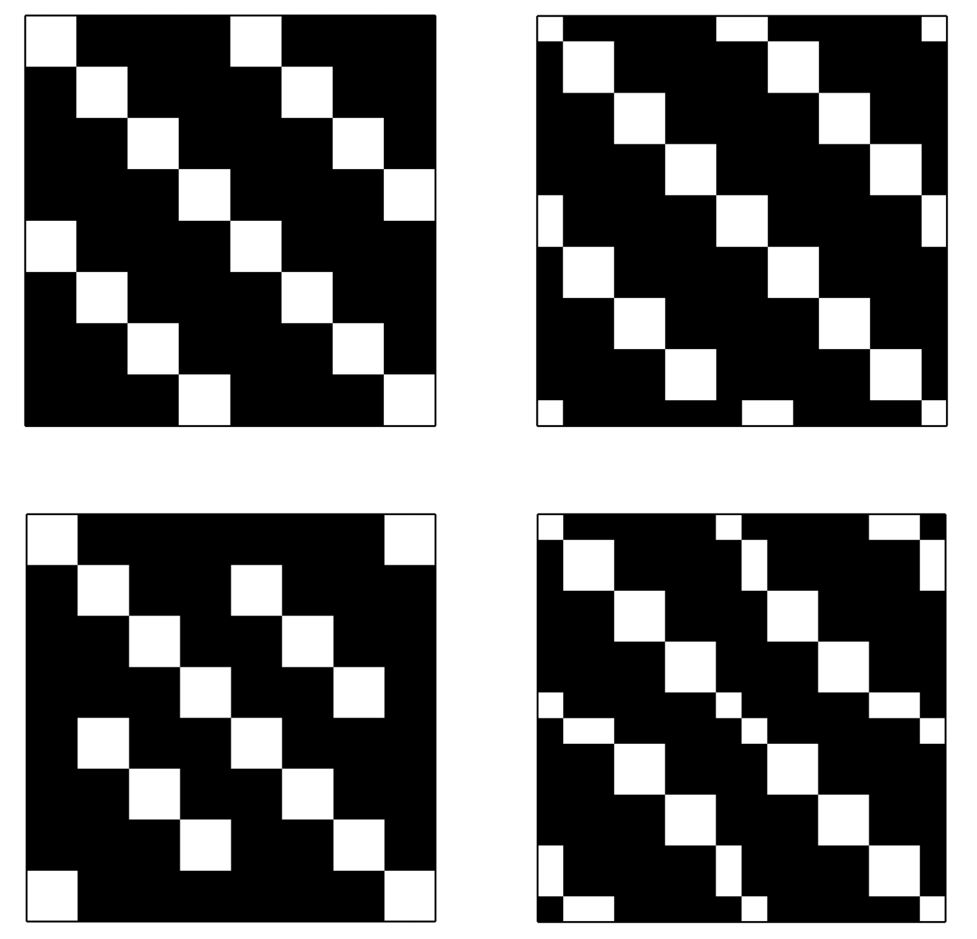


**Fig. S2 The 16 ×16 idealized matrices illustrating spatial representations.** These matrices were used to examine the neural effects of location and direction separately, while controlling for overlapping spatial factors. To isolate a specific representation (e.g., direction), we compared the average similarity within white cells against black cells. Top row shows egocentric representations of the location and direction. Bottom row shows allocentric representations of the location and direction. In these matrices, white cells indicate that the pairs of objects from odd and even runs share the same spatial properties (e.g., same location or direction), while black cells indicate that the pairs of objects differ in spatial properties (e.g., different locations or directions).

**
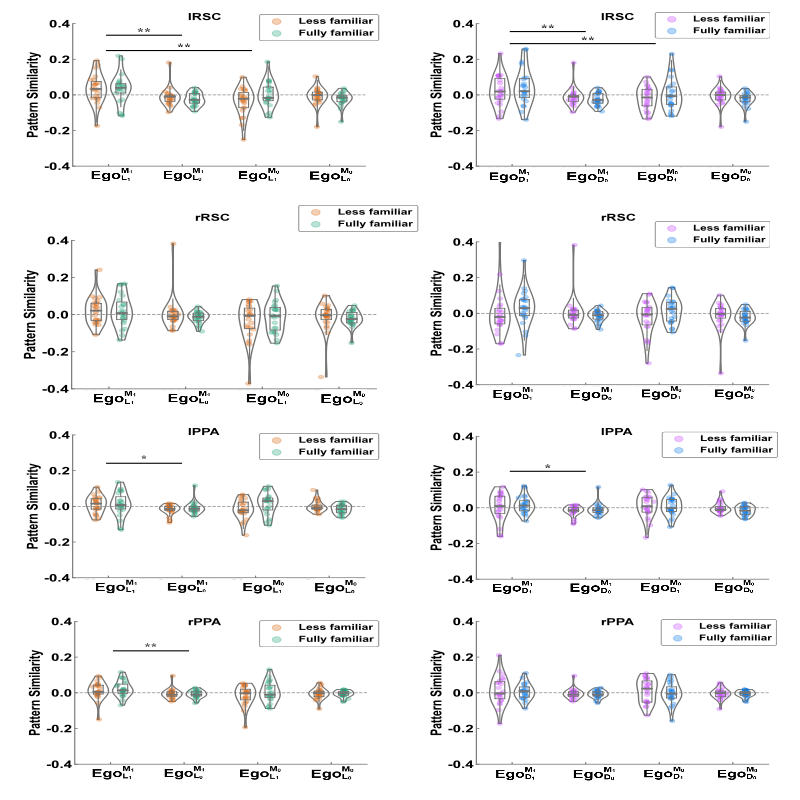
**

**Fig. S3 Neural pattern similarity for unilateral ROIs.** In addition to the main analyses on the bilateral ROIs (RSA and PPA) described in the main text, we conducted exploratory analyses to evaluate whether the egocentric representations of location and direction observed in the left and right hemispheres separately. The top row shows the results for location encoding, and the bottom row shows the results for direction encoding. Each point represents one subject. *, *p* < .05; **, *p* < .01. Abbreviations: Ego, egocentric; Allo, allocentric; M, museum; L, location; D, direction. The numbers 1 and 0 indicate whether the attribute was the same or different.


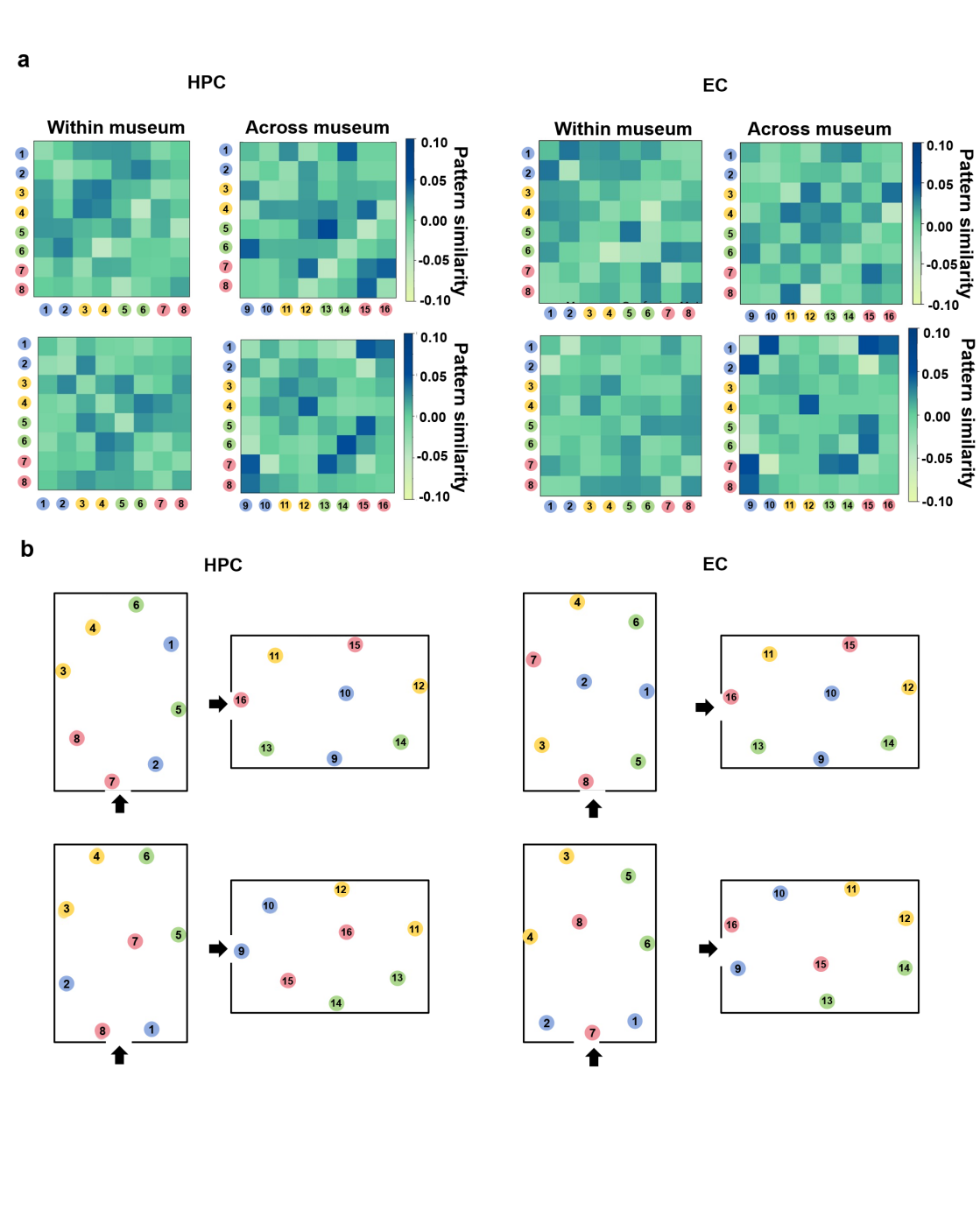


**Fig. S4 Pearson correlation matrices and reconstructed spatial layouts for the hippocampus (HPC) and entorhinal cortex (EC).** (a) Average Pearson correlation matrices between objects within the same museum (left column) and across different museums (right column) for the HPC and EC. The top row represents the less familiar stage, and the bottom row represents the fully familiar stage. (b) Reconstructed spatial layouts for the HPC and EC based on the multidimensional scaling (MDS) results. (Top row) for the less familiar stage. (Bottom row) for the fully familiar stage. The colored circles are the same as those in Fig. 4, representing different locations in the museums.
